# Perceptions of primary health care professionals from Brazil about the food and nutrition monitoring system

**DOI:** 10.1371/journal.pone.0311732

**Published:** 2024-10-25

**Authors:** Brena Barreto Barbosa, Maria Soraia Pinto, Claudia Machado Coelho Souza de Vasconcelos, Alanderson Alves Ramalho, Bartira Mendes Gorgulho, Jackeline Christiane Pinto Lobato, Luiza Jane Eyre de Souza Vieira, Patrícia Simone Nogueira, Paulo Rogério Melo Rodrigues, Ricardo José Soares Pontes, Rogério Lessa Horta, Valéria Troncoso Baltar, Antônio Augusto Ferreira Carioca

**Affiliations:** 1 University of Fortaleza, Health Sciences Center, Fortaleza, Ceará, Brazil; 2 State University of Ceará, Graduate Program in Nutrition and Health, Fortaleza, Ceará, Brazil; 3 Federal University of Acre, Graduate Program in Collective Health, Rio Branco, Acre, Brazil; 4 Faculty of Nutrition, Federal University of Mato Grosso, Cuiabá, Mato Grosso, Brazil; 5 Collective Health Institute, Fluminense Federal University, Niteroi, Rio de Janeiro, Brazil; 6 Department of Community Health, Federal University of Ceará, Fortaleza, Ceará, Brazil; 7 FEEVALE University, Academic Masters in Psychology, Novo Hamburgo, Rio Grande do Sul, Brazil; Fundacao Oswaldo Cruz Instituto Rene Rachou, BRAZIL

## Abstract

The main factors related to the lack of coverage in Health Information Systems are concentrated in the scarce and incipient training of health professionals regarding the collection and typing of data, as well as the importance of using information. The aim of this study was to analyze the perceptions of primary health care professionals from Brazil about the functioning of the Food and Nutritional Monitoring System (SISVAN). Multicentric qualitative study, carried out with 38 health professionals in Basic Health Units (BHU) in five regions around the country. Data collection took place through interviews, which were submitted to content analysis, using the thematic modality. The treatment of the results and interpretation of the themes were carried out using the theoretical framework of the philosopher Michel Foucault. Four themes emerged: (Lack of) knowledge of SISVAN; SISVAN and the conditional income transfer program; Difficulties in the execution and use of SISVAN information; and Strengths. Some of the interviewees recognized the purpose of SISVAN’s functioning. The collection of anthropometric data was related to the conditions of the Bolsa Família Program. Ignorance of the system and/or limited perception emerged as obstacles in the operability, use and quality of the data. The participants recognize that professional training is necessary to optimize the strengths of the system.

## Introduction

Food and Nutritional Monitoring (FNM) encompasses continuous data collection and analysis actions that enable the routine evaluation and organization of nutritional care of users in the Unified Health System (SUS), thus, identifying priorities according to the population’s food and nutritional situation [1]. The FNM, in Primary Health Care (PHC), includes anthropometric assessment and data on the food consumption of SUS users in all stages of their life [2].

Data on the nutritional status and food consumption markers of the population assisted in the PHC are available on the online platform of the Food and Nutritional Monitoring System (SISVAN), SISVAN Web [3]. The platform stores the records inserted in the *Bolsa Família* Program Management System, in the e-SUS APS and in the SISVAN itself, generating reports to support the planning of care and decision-making by managers in regard to the nutritional health of the population [4].

Since the implementation of its online platform in 2004, SISVAN has not been used to its full potential. The low percentages of system coverage result in the production of insufficient data for its final objective to be achieved, resulting in the system being little used by management and health professionals [5]. These professionals are indispensable in the operationalization of SISVAN, as the main factors related to its lack of coverage are concentrated in the scarce and incipient training of its workforce in regard to the collection and entering of data as well as the importance of using the information [6].

There are few studies about the perception of health professionals about the functionalities of SISVAN. Most available surveys are limited to specific professional categories [7] or the scope is restricted to the municipal or state level [8,9]. These studies recognize the importance of the system, its fragility and lack of structure, which compromises the monitoring of the main nutritional problems of PHC users. There are still questions to be explored in depth regarding the relationship between the different professional categories that work in PHC in the different socioeconomic contexts of the macro-regions of Brazil and the SISVAN, in which the findings can support the creation of strategies to mitigate the challenges that interfere with its functioning and data quality.

Given the above, this article analyzes the perceptions of primary health care professionals from Brazil about the functioning of the SISVAN.

## Methods

This is a study with a qualitative approach that is part of the multicenter research. One of its objectives was to analyze the main challenges of SISVAN from the perspective of managers, health professionals, and users in five regions of Brazil.

The Consolidated Criteria for Reporting Qualitative Research (COREQ) checklist was used as methodological guidance with the aim of improving the structure and credibility of this study [10].

The research team consisted of nutritionists, nurses, and physicians, as well as specialists in public health, nursing, and psychology. Participants were included in the study for convenience, in a consecutive and non-probabilistic way, using the principles of intentional sampling. The sample closure was due to content saturation, independent of the municipalities participating in the research. Therefore, the inclusion of participants was interrupted when the researcher from each study center identified that sufficient content was obtained, since redundancy of information was reached [11].

Data collection was carried out by postgraduate students in health who received theoretical and practical training in standardizing procedures prior to the role play interviews [12].

Data collection took place from September 2020 to June 2022, coinciding with the COVID-19 pandemic. The scenarios were the Basic Health Units (BHU) of the following cities: Cuiabá (MT), Fortaleza (CE), Novo Hamburgo (RS), Rio Branco (AC), and Niterói (RJ). In each municipality, two BHUs were selected, one located in a neighborhood with a low Human Development Index (HDI) and the other in a neighborhood with a high HDI, to assess different socioeconomic contexts. The contextual characteristics of the locations are described in Table 1.

**Table 1:** Contextual characteristics of the study scenarios.

Contextual characteristics	Cuiabá (MT)	Fortaleza (CE)	Novo Hamburgo (RS)	Rio Branco (AC)	Niterói (RJ)
Coverage of nutritional status in SISVAN Web in adults in 2019, % ^(a)^ [13]	11,1	20,0	2,7	7,7	6,8
Overweight in adults in the municipality, % ^(a)^ [13]	70,0	71,9	76,5	68,3	69,3
Municipal Human Development Index, 2010 ^(b)^ [14]	0,785	0,754	0,747	0,727	0,837
Gross Domestic Product per Capita (BRL), 2019 ^(b)^ [14]	40.199	25.254	39.592	24.448	90.643
Total population ^(c)^ [15]	623.614	2.703.391	247.303	419.452	511.786
Population covered by family health teams, % ^(c)^ [15]	42,0	54,6	43,0	62,9	64,7

(a) SISVAN Web: Module for generating public reports: Nutritional status of individuals monitored by period, stage of the life cycle and index, 2019.

(b) Brazilian Institute of Geography and Statistics. Population report by city.

(c) E-Manager Primary Care: Information and access to Primary Care systems, 2019.

The interviews were conducted using a semi-structured script. The interviewers had no previous contact with the interviewees and explained the research objectives during the interviews. The guiding questions of the script were: “What does the Food and Nutrition Monitoring System (SISVAN) represent for your professional practice?”; “How are SISVAN data collected and used in the daily routine of health services and in the professional practice?”; “What are the strengths in relation to SISVAN?”; “What are the weaknesses in relation to SISVAN?”; “What is your perception about the use and user support of SISVAN?”; “Could you point out some challenges of SISVAN?”; and “Based on these challenges, could you tell us about possible recommendations to improve them?”.

The interviews were carried out individually in a reserved place or virtual environment (via the Google Meet communication tool), lasted an average of one hour and were recorded in audio after signing/accepting the Informed Consent Form. To ensure anonymity, the remarks were identified by the initial letter of the professional category, using N for nurse, NT for nursing technician, D for doctor, NUT for nutritionist, PH for pharmacist, CHA for community health agent, and OHT for oral health technician, followed by increasing numbers following the order of participation, as in the examples: (N1, N5, NT2…).

The interviews were transcribed and submitted to content analysis in the thematic modality [16], which consists of three steps: pre-analysis, material exploration, and treatment of results and interpretation. In the pre-analysis, an in-depth reading of the transcribed statements was carried out to systematize the initial impressions of the content. The material exploration stage consisted of coding, by reducing the text to expressions or words and subsequent aggregation into thematic categories. The categories were not defined a priori and were identified according to the most evident theme in each grouping. In the treatment of the results and interpretation, the analysis of the information obtained was carried out based on the theoretical ideas of the philosopher Michel Foucault and other references available in the literature were used to provide adequate support to the inference of the exposed social and symbolic contexts.

The research was funded by the National Council for Scientific and Technological Development—CNPq (n° 26/2019) and approved by the Research Ethics Committee of the University of Fortaleza, according to ruling n° 4.348.452 and CAAE 31540320.9.1001.5052, as well as the four other participating institutions.

## Results and discussion

38 health professionals from the five regions of the country took part in the study. Participants were predominantly female, aged between 33 and 41 years old, had completed higher education, and had worked in PHC ≤10 years. The professional categories included: nursing technicians (9), community health agents (9), nurses (7), physicians (7), nutritionists (3), pharmacist (1), oral health technician (1), and pharmaceutical technician (1) (Table 2).

**Table 2:** Individual characteristics.

Individual characteristics	Cuiabá (n = 7)	Fortaleza (n = 8)	Novo Hamburgo (n = 8)	Rio Branco (n = 8)	Niterói (n = 7)	
Women	7	7	7	2		5
Age, mean (standard deviation)	42 (4,7)	41 (9,1)	40 (4,4)	33 (7,2)		45 (12,0)
Education level, higher	1	4	2	7		5
Time working in PHC, ≤ 10 years	2	6	3	6		4

Results expressed in absolute values.

The systematization of the categories to be described does not express autonomy in relation to the other categories presented; it is an exhibition resource that seeks to reveal the interweaving between the categories. In this sense, four thematic categories were established: 1) (Lack of) knowledge of SISVAN; 2) SISVAN and the conditional income transfer program; 3) Difficulties in execution and use; and 4) Strengths of SISVAN.

### (Lack of) knowledge of SISVAN

The definition and recognition of SISVAN presented a variation that ranges from unfamiliarity with the system to understanding it and its proper use. Some professionals claimed not to know the system, the statements below illustrate how, despite the existence of SISVAN, certain professionals are still unaware or do not have a broad understanding of the objectives of their actions and connections with other actions.

“So, in my daily life, in the SUS, you know, as a family and community doctor, I really didn’t know about SISVAN, that we could use this system” (D1).

According to Foucault [17], knowledge, power and understanding are interrelated. Power, which is exercised in human relationships, is productive, builds knowledge, forms discourse. The discourse that establishes society, in a privileged way, is that of the one who holds knowledge, which, when exercised, constitutes new power relations. Even the lack of knowledge reveals a social position, since there is no such thing as neutral knowledge.

Lack of knowledge about SISVAN was present in the interviewees’ speech, regardless of the level of education and professional category, with the exception of nutrition and nursing professionals. Nutritionists and nurses are primarily responsible for entering, collecting and analyzing data and for carrying out SISVAN actions [5].

“SISVAN… the nutritionist types it in, we use the e-SUS system to post the patient’s anthropometric assessment” (NT1).

Knowledge as a form of power and generator of truths has as one of its results the conservation of norms that induce and reproduce previous discourses and knowledge, already established [17]. The association of nutrition professionals with the functioning of the system is reinforced by legislation in Ordinance No. 2,246/2004, which recommends that nutritionists use the system to coordinate their activities [18]. Although this is an important profession in the operation of SISVAN, the FNM must be exercised by all professionals and managers who work in a PHC [2].

Even among professionals who claimed to know about SISVAN, answers were found that revealed a partial, fragmented and restricted understanding of the system, but that it had a strong relationship with the maternal and child population.

“[…] I know it is of fundamental importance. Because we work with pregnant women, children […] plus the children in terms of guidance, food, right?” (N2).

This association of SISVAN with children and pregnant women can be attributed to the fact that more than 85% of SISVAN data come from the *Bolsa Família* Program, (PBF), a nationwide social welfare initiative. PBF is a conditional cash transfer program aimed at supporting families living in poverty or extreme poverty while expanding their access to education and health services [19]. This will be further explored in the following topic.

### SISVAN and the conditional income transfer program

The non-recognition of SISVAN as a Health Information System (HIS) for the entire Brazilian population was evidenced, since there was the belief that it is only part of the PBF, as shown below.

“[…] the Bolsa Família was something that it [Bolsa Família] could have been stronger, it [Bolsa Família] could have come with a program within the health system” (N4).

One of the objectives of the PBF is to improve the nutritional status of the beneficiaries, therefore, one of its conditions in the health sector is the registration of anthropometric data of children under seven years of age and pregnant women during prenatal care [20]. This monitoring information of the nutritional status, which is included in the PBF Management System, migrates to SISVAN every six months [3], reinforcing coverage in these stages of life and justifying the connection of SISVAN to this population [19].

The presence of users at the health unit for the collection of SISVAN anthropometric data was identified as dependent on granting PBF benefits.

“The really bad thing is that many receive *Bolsa Família*, but they don’t come and get weighed […] when they are afraid of losing the benefit, *then* they come” (NT2).

Monitoring PBF conditions works as a stimulus for the use of health services for people in socially vulnerable situations [3]. In a critique of social medicine, Foucault [17] analyzes the existing power relations between the government and the beneficiaries of assistance systems, who must submit to various health controls, reproducing the concept of conditional assistance. This biopower, which regulates life, works not only as a way to help vulnerable populations meet their health needs, but also as a means of ensuring productivity and economic protection [21].

With regard to the execution of FNM actions, these end up being reduced to the recording of information, often in a way that is not linked to the right to access comprehensive health care [22]. In a study carried out with PHC professionals in the city of Rio de Janeiro, this practice was referred to as a “weighing and measuring culture”, referring to the measurement of anthropometric data without carrying out a nutritional diagnosis to identify cases of risk and intervention [8].

The lack of knowledge in regard to the purpose of monitoring the conditions for beneficiaries suggests an inefficient communication between professionals and users. Communication in health services can be hampered by social and cultural differences between workers and the population, this can be a result of training that favors scientific knowledge and an inadequacy of language in care situations [23], as evidenced in the statement below.

“[…] many of them don’t even know why they are here, they don’t know why it matters, they only come because they want the money” (CHA2).

This difference of knowledge, in which one type of knowledge is disqualified by the knowledge of the other, Foucault [17] characterizes as dominated knowledge. Understood as hierarchically inferior, it is common knowledge, which is present, for example, in the relationship between the “patient” and the health professional. Furthermore, there may be difficulties in communication between professionals, which weakens health actions and can harm users [24]. The very limited understanding of SISVAN and its purposes, identified in the professionals’ remarks, can be a relevant factor in the beneficiaries’ lack of knowledge.

### Difficulties in execution and use

This thematic category presents the difficulties that the participants pointed out in the execution and use of SISVAN.

“These are challenges, the internal organization of the unit, the lack of human resources […]. We have nursing technicians, but they are not always free to do the weighing” (OHT1).

“[…] we only have this computer, but the real problem […] is that the system is very slow” (CHA2).

The difficulties reported by the professionals coincide with the main obstacles related to the operation of SISVAN previously identified in the literature, suggesting that there were no important changes in this scenario. These difficulties include: insufficient and poorly maintained computers and anthropometric equipment, work overload, lack of trained professionals to collect and enter data, insufficient number of nutritionists, professional turnover, slow internet, and when the SISVAN system was “down” [5,6].

The low coverage of the system also contributes to the underuse of data in the organization of food and nutrition actions.

“We end up not using SISVAN to carry out our work, […] because if you look at the amount of data you have in SISVAN it is illusory, it is a very small number of patients” (NUT2).

In a study carried out with municipal officials from SISVAN in Minas Gerais, it was identified that most professionals and managers do not use the system’s information for planning or managing and evaluating food and nutrition [5]. As identified in the present study, the low coverage of the system is one of the factors that influence this non-use, showing that SISVAN is used more as a data storage system than for information generation [25].

As the data collection period for this study coincided with the COVID-19 pandemic, this situation was mentioned as an impediment to the execution and use of the system. To face the pandemic scenario, the BHUs had to reorganize their work processes and adapt their offer of services [26].

“[…] in the time of the pandemic, we were completely overloaded with respiratory symptoms, ultimately we weren’t doing the Bolsa Familia” (N3).

The professionals’ own lack of knowledge, explored in the first thematic category, and the lack of training to implement and use the system were perceived as an obstacle to the functioning of SISVAN.

“[…] there are many professionals who don’t know what SISVAN is. So I think that’s it, it’s having training, it’s how to use the tool” (E4).

The training of SISVAN professionals is one of the directions to overcome difficulties and improve the functioning of the system [27]. In addition, greater investment by public management in structural conditions and improvement in the generation of information from the monitoring system itself is perceived.

Monitoring here can be understood as being more than the continuous monitoring of the food and nutritional status of users by health professionals. It is understood that there is a constant observation that starts from managers to professionals, which, when analyzed according to Foucault’s concept of monitoring, is manifested in the form of a discipline that demands the continuous recording and transfer of information that comes from the ground up [28].

### Strengths as a monitoring system

In this category, the potential of SISVAN for the organization of nutritional care and decision-making in health was mentioned.

“[…] it is very important in this population’s dietary control […] we manage to find many diagnoses, identify the reason for some health conditions in that territory” (N5).

The potential of SISVAN is associated with the provision of support in decision-making by professionals and management, allowing for the identification of population groups at greater risk of nutritional problems and contributing to the planning of priority actions [3,5]. However, the low coverage of the system, especially among adolescents, adults, and the elderly, still does not allow its use to support the structuring and reorientation of public policies [19].

An important point that was present in the remarks of the interviewees are the benefits of FNM to the population.

“[…] the population, right. It gets feedback […] we manage to control breastfeeding, weight, right, of children, pregnant women, prenatal care” (N2).

The advantages are mainly recognized for mothers and children, as they are the priority population to be monitored by the PBF and represent the highest percentage of system coverage [19]. Monitoring this population is essential, however, it is worth mentioning that the purpose of SISVAN is to monitor all stages of life [3].

It is noticed that many professionals do not enter information into the system, beyond the PBF conditions, due to lack of understanding of the relevance or lack of training for use, impacting the coverage and quality of data. Furthermore, they do not use the system as a source of information due to its low coverage and limited management competence, creating a vicious circle that prevents SISVAN from being used to its full capacity.

In this process, the user is seen, but not seen. It is the object of information, but never the subject of communication. Similar to what Foucault [28] reports is happening in schools, hospitals, and even prisons, where the constant monitoring of individuals in these spaces is a manifestation of power that is predominantly exercised unilaterally and has a biopolitical and economic purpose. Greater communication and interaction between users, professionals, and managers could enhance the actions of SISVAN, so that they were carried out based on their importance for improving health, and not just as a work chore or assistance conditionality.

Our limitation is the period of data collection, which involved more than 20 months and coincided with the Covid-19 pandemic. These factors may have influenced the answers, the search for health units and the work processes within them. We emphasize that, although health professionals from only five municipalities participated in the study, we sought to ensure distinct socioeconomic contexts in all Brazilian macro-regions.

## Conclusion

Knowledge about SISVAN among health professionals was not unanimous, but was greater among nutritionists and nurses. Ignorance of the system and/or limited understanding of it emerged as the main obstacle to its operation, interfering with its execution, use, and data quality.

The presence of the PBF beneficiary population for data collection at the basic health unit was perceived as being out of context and dependent on the PBF financial conditions. In the perception of professionals, a simplistic view of health conditions prevails as a way to guarantee that the users receive the benefit.

Professionals recognized that the system was underused and that the low coverage acted as impediments in making decisions that facilitate the organization of nutritional care, as well as pointing out structural weaknesses, including the information system itself. They also identified SISVAN’s potential, such as the benefits for monitoring the health of the population, in addition to pointing out the need for professional training to strengthen the system, evidencing a critical perspective that could be used in directing solutions to the difficulties shown.

## Supporting information

S1 Checklist COREQ (COnsolidated criteria for REporting Qualitative research) checklist. (PDF)
