# Correction: Perceptions of primary health care professionals from Brazil about the food and nutrition monitoring system

**DOI:** 10.1371/journal.pone.0320590

**Published:** 2025-03-18

**Authors:** Brena Barreto Barbosa, Maria Soraia Pinto, Claudia Machado Coelho Souza de Vasconcelos, Alanderson Alves Ramalho, Bartira Mendes Gorgulho, Jackeline Christiane Pinto Lobato, Luiza Jane Eyre de Souza Vieira, Patrícia Simone Nogueira, Paulo Rogério Melo Rodrigues, Ricardo José Soares Pontes, Rogério Lessa Horta, Valéria Troncoso Baltar, Antônio Augusto Ferreira Carioca

The affiliation for the 13^th^ author is incorrect. Antônio Augusto Ferreira Carioca is not affiliated with #2 but with #1: University of Fortaleza, Health Sciences Center, Fortaleza, Ceará, Brazil.
